# A Systematic Review of the Literature Examining the Effects of Cigarette Smoke and e-Cigarette Vapor on the Virulence of Human Pathogenic Bacteria

**DOI:** 10.3390/ijerph191912518

**Published:** 2022-09-30

**Authors:** Kamal Bagale, Ritwij Kulkarni

**Affiliations:** Department of Biology, University of Louisiana at Lafayette, Lafayette, LA 70504, USA

**Keywords:** cigarette smoke, e-cigarette vapor, smoking, vaping, respiratory pathogens, virulence, transcriptome

## Abstract

The bioactive chemicals in cigarette smoke (CS) and e-cigarette vapor (EV) may affect pathogenic bacteria in the nasopharyngeal microflora, which may have implications on the pathophysiology of respiratory infections in cigarette smokers and e-cigarette users. In this systematic review, we seek to synthesize the research evidence supporting this hypothesis. To address the central research question, “*what is known from the published, peer-reviewed literature about the effects of cigarette smoke or e-cigarette vapor exposure on the physiology of human pathogenic bacteria?*”, we screened the PubMed^®^, Web of Science^TM^, and ScienceDirect databases for reports examining the virulence characteristics and gene expression in human pathogenic bacteria exposed to either CS or EV. The principal conclusion from our analysis is that exposure to either CS or EV induces the virulence of respiratory pathogenic bacteria in a strain-dependent manner, which may in turn facilitate respiratory infections in cigarette smokers and e-cigarette users. In addition, we present evidence that nicotine and reactive oxygen species are the main chemicals responsible for CS/EV-mediated alterations in bacterial physiology. We note limitations that this review does not examine reports describing the alterations in host respiratory physiology or nasopharyngeal dysbiosis caused by CS/EV exposure. Future research to determine whether CS/EV-mediated augmentation of bacterial virulence indeed plays a role in human respiratory tract infections is warranted.

## 1. Introduction

Exposure to cigarette smoke (CS) is an established risk factor for respiratory infections such as rhinosinusitis, bronchitis, and pneumonia [1] as well as for periodontal disease [2]. The gas and tar phases of CS contain bioactive chemicals acrolein, carbon monoxide, nicotine, nitrogen oxide, cadmium, acetaldehyde, formaldehyde, ammonia, and reactive oxygen species (ROS), which induce mechanical and structural changes in the respiratory tract, affect respiratory immunity, and in turn predispose CS-exposed individuals to respiratory infections [3]. Due to the adverse health effects of CS exposure, various government and non-government agencies have led decades-long efforts to promote smoking cessation. This approach has been successful as evidenced by a steep drop in the prevalence of cigarette smoking among adults ≥ 18 years of age from ~45% in 1964 to <15% in 2020 in the US [4]. However, in the last 15 years, the e-cigarette has gained in popularity, especially amongst youth and middle school- and high school-going teenagers. The e-cigarette is a handheld device that heats a mixture of humectants, propylene glycol and vegetable glycerin, flavoring chemicals, and nicotine, called the e-liquid, to generate aerosolized vapor [5]. The e-liquid is commercially sold in a specific, pre-mixed composition containing various concentrations of nicotine or as a customizable kit containing separate vials of humectants, flavoring chemicals, and nicotine [6]. The e-cigarette was originally introduced as a safe alternative to traditional cigarettes that can be used as a smoking cessation device [7]. While e-cigarette vapor (EV) contains far fewer chemicals than CS, mounting scientific evidence suggests that the chronic exposure to EV can adversely affect host respiratory health. In 2019, there was an outbreak of e-cigarette, or vaping, product use-associated lung injury (EVALI) in the US, resulting in 2807 hospitalizations and 68 deaths (www.cdc.gov/EVALI, accessed on 13 August 2022). 

Since the resident microflora of the respiratory tract and the mouth consists of both commensal microorganisms and pathogens, CS/EV may directly affect pathogen virulence. In this systematic review, we sought to synthesize results from research reports confirming this hypothesis. To answer the central research question “*what is known from the published, peer-reviewed literature about the effects of cigarette smoke or e-cigarette vapor exposure on the physiology of human pathogenic bacteria?*”, we analyzed reports where bacterial pathogens pre-exposed to either CS or EV were examined using in vitro and in vivo assays of virulence and gene expression analysis. In the narrative, after describing the protocol used for screening published literature, we present sections describing the effects of CS/EV exposure on bacterial growth, virulence factors (biofilm formation and adherence), gene expression, and conclusions. As limitations, we note that this review does not examine the research evidence that the exposure to CS/EV mediates respiratory and oral dysbiosis and significantly alters the respiratory immunity against pathogens [1,8,9].

## 2. Materials and Methods

**Figure 1:**
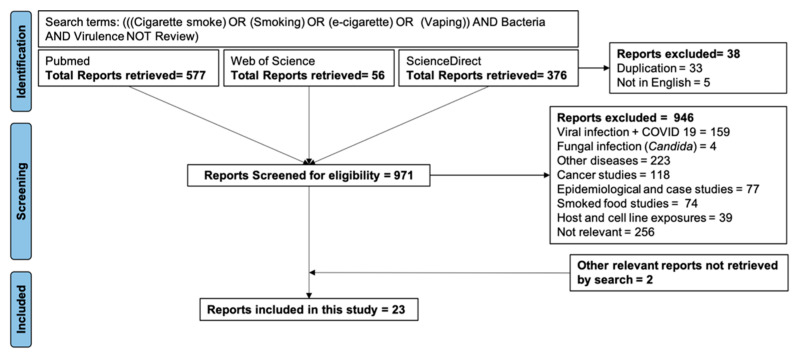
PRISMA criteria for selecting studies for review.

We searched the PubMed^®^, Web of Science^TM^, and ScienceDirect databases using the terms (((cigarette smoke) OR (smoking) OR (e-cigarette) OR vaping)) AND bacteria AND virulence NOT review). The first search was conducted on 30 December 2021 while the latest search was conducted on 7 September 2022. We retrieved a total of 1009 articles, of which 33 were duplicates between three databases and 5 were not in English. After accounting for the duplicates and excluding non-English articles, the remaining 971 were independently screened by the two authors. The exclusion and inclusion criteria used for screening the articles are shown in Figure 1. The exclusion criteria were reports describing the effects of CS and/or EV on viral and fungal infections, non-infectious diseases, and cancer, epidemiological and case studies, reports describing the effects of smoking meat on food-borne pathogens, studies examining the effects of CS/EV on pathogen-specific immune defenses elicited by cell lines or by vertebrate hosts, and reports not examining the effects of CS/EV on bacterial pathogens. The screening step retrieved 21 reports congruent with the primary objective of our research question. To these, we added 2 reports missed by the original database searches. 

To describe CS and EV formulations in different studies, we have consistently used terms as defined below; however, these terms do not always match those used in the original publications: (i) cigarette smoke extract (CSE) and e-cigarette vapor extract (EVE) refer to aqueous formulations wherein cigarette smoke (from a defined number of cigarettes) or e-cigarette vapor was bubbled through a specific amount of culture medium or PBS or saline under vacuum. Here, EVE^NIC+^ refers to EVE from nicotine-containing e-liquid and EVE^NIC-^ to EVE from nicotine-free e-liquid. The cigarette brands and the flavoring and the amount of nicotine in EVE may be different between studies. (ii) CS or EV refer to experimental setups where bacteria growing on solid agar plates or liquid nutrient-rich media were placed in an exposure chamber through which CS or EV is drawn by vacuum. Here, EV^NIC+^ and EV^NIC-^ refer to EV from either nicotine- containing or nicotine-free e-liquids. (iii) Cigarette smoke condensate (CSC) is a commercial formulation (Murty Pharmaceutical, Lexington, KY, USA), where CS was passed through a filter and the particulate matter collected on the filter is dissolved in 40 mg/mL DMSO.

## 3. Results

The main results from the peer-reviewed reports are summarized in Table 1.

### 3.1. The Effects of CS and EV Exposure on the Bacterial Growth

The effects of CS/EV exposure on bacterial growth are dependent on the CS/EV formulations used for exposure, length of exposure, and the bacterial species (Table 1). CSE generated by drawing smoke from 1 cigarette in 10 mL culture medium slowed the growth of MRSA strain USA300 [15], *P. aeruginosa* [23], and a panel of hospital-acquired (HA-MRSA), community-acquired (CA-MRSA), and laboratory MRSA strains (Newman and SH1000) [11] in a dose-dependent manner. The ROS and nicotine were identified as the principal chemicals responsible for CS/EV-mediated inhibition of bacterial growth based on the following observations: (i) CSE pre-treated with ROS-inhibitor N-acetyl cysteine (NAC) did not inhibit MRSA growth [11]; (ii) compared to their WT counterparts, the growth of MRSA mutants lacking activity of either one of the two oxidative stress response genes, catalase (*katA*) or alkyl hydroxyperoxidase (*ahpC*), was significantly more affected by CSE [11]; (iii) in a dose-dependent manner, nicotine decreased the growth of *S. aureus* [10,14] and *P. gingivalis* [26]. Notably, MRSA growth was completely suppressed in the presence of individual e-cigarette components, polyethylene glycol and vegetable glycerin [14]. In contrast, Gilpin et al. did not report reduction in the growth of *H. influenzae*, *S. aureus*, *S. pneumoniae*, or *P. aeruginosa* upon cultivation for 48 h in either CSE or EVE^NIC+^ [12]. Although in this case, CSE and EVE^NIC+^ were generated by drawing either smoke from 4 cigarettes or vapor from e-liquid containing 10 mg/mL nicotine for 20 min into 100 mL culture medium, respectively, which may have diluted CS or EV^NIC+^ chemicals [12]. Researchers have also noted that exposure to 160 µg/mL CSC for 16 h did not affect the viability of *S. pneumoniae* [20], exposure either to CSE or to nicotine (6 µg/mL) did not affect the growth of *P. gingivalis* [24,27]. Exposing *S. mutans* to CS or EV^NIC+^ or EV^NIC-^ in 15 min bursts twice a day also did not affect *S. mutans* growth on BHI [22].

### 3.2. The Effects of CS and EV Exposure on Bacterial Biofilm Formation

Biofilms are comprised of microbial cells bound by an extracellular matrix which promotes survival and growth [28]. The biofilm extracellular matrix forms a protective barrier that prevents penetration of immune cells and antibiotics, and facilitates horizontal gene transfer events that can increase the virulence of human pathogens [29]. A biofilm forms in four sequential steps: (i) initial adhesion to a surface facilitated by extracellular adhesins; (ii) formation of microbial aggregates within an extracellular matrix made of proteins, nucleic acids, and polysaccharides; (iii) maturation and biofilm growth; and (iv) dispersal of small biofilm clumps to form secondary biofilms [30]. Biofilms formed by *P. aeruginosa*, *E. coli*, and *S. aureus* have been linked to human respiratory diseases [31]. 

The in vitro exposure to either CS or EV augments biofilm formation by microbial pathogens on various abiotic surfaces as summarized in Table 1. Overall, these results established that 18–24 h-long exposure to varying concentrations of either CSE or EVE results in significant and dose-dependent augmentation of biofilms on the polystyrene surface by various respiratory pathogens: (i) overnight exposure to CSE induced biofilm formation by methicillin-resistant (MRSA) and methicillin-sensitive (MSSA) strains of *S. aureus* [11,16], *S. pneumoniae* [12,18], *P. aeruginosa* strains PAO1 and ATCC 27853 [12,23], and by *P. gingivalis* [25]; (ii) acute (3 h) exposure to CSE significantly augmented biofilms by sinonasal bacteria from smokers with chronic rhinosinusitis, although the biofilm formation by sinonasal bacteria from non-smokers with chronic rhinosinusitis was unaffected by CSE exposure [32]; (iii) EVE^NIC+^ augmented biofilm formation by MRSA [14] and *S. pneumoniae* strain TIGR4 [18]; (iv) overnight exposure to CSC augmented biofilms by a variety of clinical and laboratory isolates of *S. pneumoniae* [19,20,33]; (v) both CS and EV also augmented biofilms of *S. mutans* seeded on either human teeth or porous collagen scaffold [22]. Notably, molecular changes underlying CS- and EV-mediated biofilm augmentation appear to be rapid and heritable because both *S. aureus* or *S. pneumoniae* pre-exposed to CSE or EVE for a short (1 to 2 h) period followed by a wash to remove traces of CS/EV and a transfer to CS/EV-free, plain culture medium in a polystyrene plate showed increased biofilm formation [16,18]. 

A number of studies have revealed the central role of ROS in CS/EV-mediated augmentation of bacterial biofilms as explained below. Both CS and EV augmented *S. aureus* biofilms via suppression of the *agr* (accessory gene regulator) two-component system of quorum sensing [14,16]. The *agr* system regulates transition between sessile and planktonic forms of staphylococci [34]: low *agr* activity promotes initial attachment and biofilm formation while biofilm detachment and dispersal are associated with the increased *agr* expression [35]. The role of ROS-mediated *agr* suppression in the augmentation of *S. aureus* biofilms in the presence of CS is emphasized by the observations that (i) CSE pre-treated with ROS inhibitor n-acetyl cysteine (NAC) neither suppressed *agr* expression nor augmented *S. aureus* biofilms [16] and (ii) CSE exposure did not augment biofilm formation either in human *S. aureus* isolates with naturally low *agr* activity or in *S. aureus* KO mutants lacking *agr* activity [11]. EVE^NIC+^ exposure also augmented *S. aureus* biofilms via suppression of *agrA* [14], although whether ROS plays a role in EV-mediated staphylococcal biofilm augmentation has not been examined experimentally. EV-augmented staphylococcal biofilms may also be structurally dissimilar from CS-augmented staphylococcal biofilms because CSE exposure has been observed to induce the expression of *icaA* encoding the polysaccharide intercellular adhesin in the biofilm matrix, while EVE^NIC+^ suppresses *icaA* [14]. In addition, CSE exposure has been observed to induce the expression of a transcriptional regulator *rbf* (required for biofilm formation) known to play a role in MRSA biofilm formation [16,36], while EVE^NIC+^ suppressed *psm* encoding phenol soluble modulins [14]. 

Nicotine has also been observed to play a role in the CS/EV-mediated augmentation of biofilms: (i) *S. pneumoniae* TIGR4 biofilm was significantly augmented by exposure to EVE^NIC+^ but not by EVE^NIC−^ [18]; (ii) exposure to 2 mg/mL nicotine augmented biofilm formation by *S. pneumoniae* TIGR4 [18] and MRSA USA300 [10]; and (iii) exposing *S. mutans* seeded on porous collagen scaffold and human teeth to CS or EV^NIC+^ induced biofilms at 24 h post-exposure, while EV^NIC−^ augmented *S. mutans* biofilms at 72 h post-exposure [22]. However, in a notable exception it was reported that exposing pre-formed *S. mutans* biofilms on human saliva-coated hydroxyapatite discs to CS in an exposure chamber reduced biofilm biomass by suppressing production of extracellular polysaccharides forming biofilm matrix although CS treatment did not induce bacterial death [21]. 

CS has been reported to augment *S. pneumoniae* (serotype 23F) biofilms via activation of *rr11* and *hk11* genes encoding the structural components of TCS 11 (two-component system 11) [19,20,33]. In contrast, in *S. pneumoniae* TIGR4, CSE was found to induce the biofilm formation without upregulating the expression of TCS11 encoding genes [18]. Thus, the role of TCS11 in CS-mediated pneumococcal biofilm augmentation appears to be strain dependent. 

In conclusion, both CS and EV rapidly augment biofilm formation on the polystyrene surface by various Gram-positive and Gram-negative pathogens in a dose- and bacterial-strain-dependent manner. Reactive oxygen species and nicotine are the main chemical drivers of CS/EV-dependent biofilm augmentation. Future experimentation is needed to determine whether CS/EV augments biofilms in the human respiratory tract.

### 3.3. The Effects of CS and EV Exposure on Bacterial Adherence

Using in vitro assays of adherence, researchers have observed that pre-exposure to CS/EV augments the adherence of *S. aureus* to various host proteins and cells by inducing the transcription of genes encoding specific surface adhesins: (i) pre-exposure to CSE induced adherence of MRSA USA300 to A549 human respiratory epithelial cells [16] and HaCaT keratinocytes [15]; (ii) CSE-exposed USA300 showed higher in vitro binding to human extracellular matrix protein fibronectin via increased expression of *fnbpA*, which encodes the surface adhesin, fibronectin binding protein A [16]; (iii) *S. aureus* mutants lacking either *fnbpA* or *srtA* (encodes sortase A enzyme that anchors surface proteins into the cell wall) did not show higher biofilm formation in presence of CSE suggesting a role for surface adhesin activity in CS-mediated biofilm augmentation [11]; (iv) in vitro exposure to either nicotine or cotinine increased adherence of oral colonizer and periodontal pathogen *P. gingivalis* to the monolayers of KB human oral epithelial cell line [37] and to human fibronectin via increased production of surface-exposed fimbria [25]. Interestingly, exposure to either CSE or EVE^NIC+^ or EVE^NIC−^ did not affect the adherence of *S. pneumoniae* TIGR4 to A549 human lung epithelial cells [17,18]. In conclusion, exposure to either CS or EV induces adherence of *S. aureus* and *P. gingivalis* to host cells and proteins while the adherence of *S. pneumoniae* or *P. aeruginosa* is unaffected by CS/EV exposure. 

### 3.4. The Effects of CS and EV on Other Virulence Characteristics

To successfully colonize and infect a host, pathogens must survive the immune onslaught. CS/EV-mediated biofilm augmentation may facilitate pathogen survival by protecting biofilm-bound pathogens from the action of immune cells and effector proteins. In addition, CS/EV exposure also induces other virulence characteristics that may help pathogens evade specific immune defenses: (i) the pre-exposure to either CSE or EVE increased the resistance of MRSA USA300 to LL-37 antimicrobial peptide (AMP) by making the bacterial surface positively charged and by increasing the surface hydrophobicity [14,15]; (ii) CSE-pre-exposure increased the ability of MRSA USA300 to evade NET (neutrophil extracellular trap)-mediated killing via upregulation of *nuc* nuclease transcription [13] although exposure to nicotine alone suppressed *S. aureus nuc* transcription [10] and CSE-exposed *P. aeruginosa* did not show increased resistance to NET-mediated killing [23]; (iii) pre-exposure to CSE significantly increased the resistance of MRSA USA300 to intracellular killing by neutrophils and alveolar macrophages of both murine and human origins which may be attributed to increased resistance to the killing by ROS and to the surface changes that make USA300 more resistant to cell lysis [13,15]; (iv) CSE-pre-exposed *P. aeruginosa* PAO1 also became resistant to neutrophil killing via increased resistance to oxidative stress but without affecting either surface charge or surface hydrophobicity [23]; (v) CSE-exposed *P. gingivalis* suppressed the production of pro-inflammatory cytokines TNF-α and IL-6 while inducing anti-inflammatory cytokine IL-10 in human PBMCs [24,25].

The pre-exposure to CSE, EVE^NIC+^, or EVE^NIC−^ did not affect the hydrophobicity of *S. pneumoniae* TIGR4 nor its susceptibility to killing by ROS [17,18]. Exposure to either CSC or CSE also suppressed the activity of pore-forming pneumolysin via reduced expression of *ply* in *S. pneumoniae* [17,18,20,30]. Gilpin et al. also reported that *S. aureus*, *S. pneumoniae*, *P. aeruginosa*, and *H. influenzae* pre-exposed to either CSE or to EVE^NIC+^ induce pro-inflammatory cytokine production by A549 epithelial cells compared to the control cells infected with medium-exposed bacteria [12]. Overall, these observations suggest that CS/EV exposure induces virulence characteristics of some pathogens providing them with a survival advantage inside the host.

### 3.5. The Effects of CS and EV Exposure on Bacterial Gene Expression

To identify specific genes underlying CS/EV induced bacterial virulence factors, researchers have compared transcription of specific genes in CS/EV-exposed bacteria and in medium-exposed controls using real-time PCR analysis. In addition, RNASeq and microarrays have also been used to compare in global transcriptomic changes in CS/EV-exposed bacteria (summarized in Table 2). Where relevant we have reviewed changes in the gene expression alongside the discussion on CS/EV-mediated changes in bacterial virulence in previous sections. In this section, we review the remaining results describing important changes in bacterial gene expression caused by CS or EV exposure.

RNASeq and microarray studies have shown that exposure to potent toxic chemicals in CS or EV induces the transcription of various genes involved in stress response, detoxification, and bacterial survival in case of *P. gingivalis*, *S. aureus*, and *S. pneumoniae* [13,17,18,24]. More specifically, CSE-exposed *P. gingivalis* showed altered expression of functional gene groups involved in oxidative stress response, DNA repair, and the virulence (surface-attached fimbriae and capsule) [24]. These results were confirmed by the proteomic analysis showing that exposure to either nicotine or cotinine alters the levels of *P. gingivalis* proteins involved in oxidative stress, virulence, and metabolism [27]. CSE exposure also altered the expression of genes involved in oxidative stress response, metabolism, amino acid synthesis, and virulence (surface proteins) in MRSA strain USA300 [13]. CSE-exposed *S. pneumoniae* showed upregulation gene encoding efflux pumps and downregulation of gene involved in the biosynthesis of fatty acid and D-alanyl lipoteichoic acid [17]. A side-by-side comparison of the effects of 2 h-long exposure to CSE, EVE^NIC+^, or EVE^NIC−^ on pneumococcal transcriptome showed that both CSE and EVE^NIC+^ altered the expression of genes involved in metabolism and stress response, although EVE^NIC+^ altered the expression of a higher number of genes (982) than CSE (264) [18]. In contrast, EVE^NIC−^ exposure altered the expression of only 14 genes all of which were involved in metabolism [18]. In summary, the transcriptome analysis shows that exposure to either CSE or EVE^NIC+^ but not EVE^NIC−^ modulated the expression of functional gene groups involved in stress response and metabolism across different pathogenic bacteria. 

The studies examining gene expression by qRTPCR have confirmed that CSE induces the expression of bacterial oxidative stress response genes such as *ahpC* (alkyl hydroxy peroxidase) and *sod* (superoxide dismutase) induced in *S. aureus* (and MRSA strains) [16] and *tpx* (thiol peroxidase) in *P. aeruginosa* [23]. The activity of oxidative stress response genes would not only facilitate bacterial survival in ROS-rich CSE but may also help these pathogens counter bactericidal ROS produced by macrophages and neutrophils. 

CSE exposure has also been reported to induce *P. aeruginosa* antimicrobial resistance via upregulation of genes encoding multidrug efflux pumps (*mexA*/*X*/*Z*, *mexEF-OprN*) or via suppression of *OprD* encoding outer membrane protein involved in carbapenem influx [23,38,39]. 

### 3.6. Examining the Effects of CS/EV on Bacterial Virulence in an In Vivo Mouse Model

Whether CS/EV-induced physiological alterations in bacteria result in the emergence of a truly hypervirulent phenotype capable of causing more severe respiratory infections can only be examined in an animal model. For this, researchers have developed an in vivo experimental setup where pathogens were first pre-exposed in vitro to CS or EV (with or without nicotine) or to medium alone (control), washed in sterile PBS to remove CS/EV chemicals, and then inoculated into the nares of anesthetized mice. The survival and respiratory bacterial burden in the infected mice were examined as the measures of bacterial virulence. Anesthetized mice aspirate bacterial inoculum into the lungs which results in acute pneumonia. As the mice are not directly exposed to CS or EV, this in vivo experimental setup effectively examines the effects of CS/EV on bacterial virulence without confounding changes in host respiratory physiology wrought by CS/EV exposure. Important observations from the experiments comparing bacterial virulence in mice intranasally inoculated with bacteria pre-exposed to either CS/EV and control mice inoculated with bacterial pre-exposed to medium alone are summarized in Table 1 and below: (i) C57Bl/6, BALB/c, and A/J mouse strains intranasally infected with MRSA strain USA300 pre-exposed to CSE showed ~10-fold higher lung bacterial burden at 24 hpi [13]; (ii) CD-1 mice infected with MRSA pre-exposed to EVE^NIC+^ showed 25% decreased survival and ~100-fold higher lung bacterial burden on day 4 post-infection [14]; (iii) CD-1 mice infected with MRSA pre-exposed to CSE also showed decreased survival up to day 4 post-infection and ~20-fold higher lung bacterial burden at 20 hpi [15]; (iv) 100% of CD1 mice infected with CSE-pre-exposed *P. aeruginosa* were dead by day 6 pi while 28% in the control group survived till day 10 pi [23]; (v) however, pre-exposing *S. pneumoniae* TIGR4 to CSE, EVE^NIC+^, or EVE^NIC−^ did not affect respiratory bacterial burden [18]. These results suggest that a short-term in vitro exposure to CS/EV can induce hypervirulence in a respiratory pathogen-dependent manner. In *Galleria mellonella* (wax moth) model of infection where reduced survival is the measure of increased pathogen virulence, the pre-exposure to either CSE or EVE^NIC+^ induced the virulence *S. aureus*, *S. pneumoniae*, and *P. aeruginosa* while in case of *H. influenzae* pre-exposure to CSE but not to EVE^NIC+^ induced virulence [12].

## 4. Conclusions

For this article, we reviewed the evidence from peer-reviewed publications examining the effects of CS and EV on the physiology of respiratory pathogens. Overall, the evidence suggests that exposure to either cigarette smoke or e-cigarette vapor affected bacterial virulence and gene expression. Moreover, CS affected bacterial gene expression and virulence more significantly than EV^NIC+^, while the EV^NIC−^ affected bacterial gene expression and virulence the least. Research also showed that the CS/EV-mediated alterations in bacterial virulence were dependent on the length of exposure and the strain of pathogen under examination and that reactive oxygen species (ROS) and nicotine were the primary chemical drivers of CS/EV-mediated alterations in the pathogen virulence. 

The reports indicating that CSE exposure induces bacterial adherence to human and murine epithelial cells by altering the surface charge on the host cells [40] or by increasing the expression of specific host protein receptors involved in bacterial adhesion [41,42] are not discussed here because this review is focused on the effects of CS/EV on bacterial pathogens. Moreover, we have also not discussed the evidence that CS exposure affects respiratory physiology by impeding mucociliary clearance of the particulate matter including microbes or by inducing inflammatory damage to the lungs [1,43]. We acknowledge these limitations and submit that the increased severity of a respiratory tract infection in a cigarette smoker or an e-cigarette user is expected to be the sum of the adverse effects of CS and EV on the physiology of both host and the pathogen. Our strategy of screening three different databases of biomedical and life sciences research literature has a limitation that it can miss relevant reports not indexed on these databases. 

Exposure to either cigarette smoke or e-cigarette vapor induces pleiotropic changes affecting the physiology of the host and the pathogens known to asymptomatically colonize the respiratory tract and the mouth. Future research is needed to examine in an in vivo model the effects of CS/EV exposure on the interactions between pathogen virulence and host immune defenses in the pulmonary milieu by the parallel transcriptomic analysis of the host and the pathogen. Additionally, it will be of significant clinical importance to determine whether CS and/or EV exposure facilitates the transition of a pathogen from an asymptomatic colonizer to a virulent cause of severe respiratory infection. Such experiments will improve our understanding of the molecular mechanisms underlying respiratory tract infections in cigarette smokers and e-cigarette users and may help us develop more effective therapeutics. 

## Figures and Tables

**Table 1:** Effects of CS/EV exposure on pathogen virulence examined in vitro and in vivo.

Exposure ^a^	Growth	Virulence ^b^			Mouse Infection ^c^			Comments ^d^	Ref.
		BF	Adh	Inv	Strain	CFU	Survival		
*Staphylococcus aureus* (including MRSA)									
Nicotine	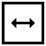	↑	↑ *	↓ ^$^	ND	ND	ND	* Adherence to polyethylene ^$^ Invasion of A549 cells	[10]
CSE	↓	↑	ND	↑ ^$^	ND	ND	ND	^$^ Invasion of bronchial epithelial cells	[11]
CSE	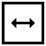	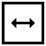	ND	ND	ND	ND	↓ ^#^	^#^*Galleria mellonella* infection model	[12]
EVE^NIC+^	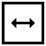	↑	ND	ND	ND	ND	↓ ^#^		
CSE	ND	ND	ND	ND	C57bl6 BALB/c A/J	↑ ↑ ↑	ND ND ND		[13]
EVE^NIC+^	↓	↑	↑ *	↑ *	CD1	↑	↓	* Adherence to, * invasion of HaCaT	[14]
CSE	↓		↑ *	↑ *	CD1	↑	↓	* Adherence to, * invasion of HaCaT	[15]
CSE	ND	↑	↑ *	ND	ND	ND	ND	* Adherence to A549 cells and to human fibronectin	[16]
*Streptococcus pneumoniae*									
CSE	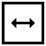	ND	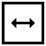 *	ND	ND	ND	ND	* Adherence to A549 cells	[17]
CSE	ND	↑	ND	ND	ND	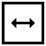	ND		[18]
EVE^NIC+^	ND	↑	ND	ND	ND	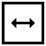	ND		
EVE^NIC−^	ND	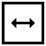	ND	ND	ND	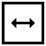	ND		
CSC	ND	↑	ND	ND	ND	ND	ND		[19]
CSE	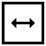	↑	ND	ND	ND	ND	↓ ^#^	^#^*Galleria mellonella* infection model	[12]
EVE^NIC+^	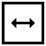	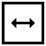	ND	ND	ND	ND	↓ ^#^		
CSC	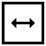	↑	ND	ND	ND	ND	ND		[20]
*Streptococcus mutans*									
CS	ND	↓ ^^^	ND	ND	ND	ND	ND	^^^ Biofilms pre-formed on hydroxyapatite discs showed reduced biomass upon CS exposure	[21]
CS	ND	↑	↑ *	ND	ND	ND	ND	* Adherence of pre-exposed *S. mutans* to human teeth was examined in vitro	[22]
EV^NIC+^	ND	↑	↑ *	ND	ND	ND	ND		
EV^NIC-^	ND	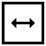	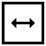 *	ND	ND	ND	ND		
*Pseudomonas aeruginosa*									
CSE	↓	↑	ND	ND	CD1	ND	↓		[23]
CSE	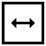	↑	ND	ND	ND	ND	↓ ^#^	^#^*Galleria mellonella* infection model	[12]
EVE^NIC+^	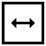	↑	ND	ND	ND	ND	↓ ^#^		
*Haemophilus influenzae*									
CSE	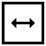	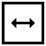	ND	ND	ND	ND	↓ ^#^	^#^*Galleria mellonella* infection model	[12]
EVE^NIC+^	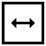	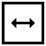	ND	ND	ND	ND	↓ ^#^		
*Porphyromonas gingivalis*									
CSE	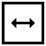	ND	ND	ND	ND	ND	ND		[24]
CSE	ND	↑	↑ *	ND	ND	ND	ND	* Increased adherence to fibronectin	[25]

Exposure ^a^ refers to CS/EV formulation or nicotine used. Virulence ^b^ shows the effects of CS/EV exposure on the virulence characteristics such as biofilm (BF) and the ability of bacteria to adhere (Adh) to or invade (Inv) cells. Mouse infection ^c^ shows mouse strain infected with CS/EV pre-exposed bacteria via intranasal route, changes in CFU (refers to pulmonary organ burden), and survival of infected mice. ^#^ Additionally, included here is a report where bacterial virulence was adjudged by the survival of ^#^ *Galleria* *mellonella* (wax moth) as indicated in Comments ^d^. 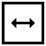 indicates no change, ↑ increase, ↓ decrease, and ND indicates not done. Comments ^d^ also define the cell lines or biotic materials used in adherence * assays and the cell lines used in invasion ^$^ assays.

**Table 2:** The effects of CS/EV in vitro exposure on bacterial transcriptome.

Pathogen	Exposure ^a^			Technique	DEG ^b^			Ref.
	Med	Form	Time		Total	Up	Down	
*S. pneumoniae*	THY ^c^	CSE	120	RNASeq	264	188	76	[18]
		EVE^NIC+^			982	500	482	
		EVE^NIC−^			14	14	0	
*S. pneumoniae*	THY ^c^	CSE	45	RNASeq	122	59	63	[17]
*S. aureus*	TS ^d^	CSE	360	RNASeq	344	204	140	[13]
*P. gingivalis*	GAM ^e^	CSE	NA	Microarray	104	58	46	[24]

Exposure ^a^ shows culture medium (Med) used for the preparation of specific CS/EV formulations (Form) and the time of exposure (in minutes). DEG ^b^ refers to differentially expressed genes; the total number of DEG and upregulated and downregulated genes are shown. THY ^c^ refers to Todd–Hewitt broth with 0.5% yeast extract. TS ^d^ refers to Tryptic soy medium. GAM ^e^ refers to Gifu anaerobic medium.

## Data Availability

An Excel data file containing a list of screened manuscripts annotated with inclusion and exclusion criteria will be made available on request.
